# Kombination stereotaktischer Radiotherapie mit Checkpointinhibitoren: Ergebnisse der CHEERS-Phase-II-Studie

**DOI:** 10.1007/s00066-023-02160-z

**Published:** 2023-10-19

**Authors:** Tobias Mohr

**Affiliations:** 1 Klinik für Strahlentherapie, Universitätsklinikum des Saarlandes, Kirrberger Str. Gebäude 6.5, 66421 Homburg/Saar, Deutschland; 2 Arbeitsgruppe junge DEGRO, Deutsche Gesellschaft für Radioonkologie e. V. (DEGRO), Berlin, Deutschland

## Hintergrund

Sowohl die Immuntherapie als auch die stereotaktische Radiotherapie sind fest etablierte Therapieoptionen in der modernen Medizin. Die hier vorliegende CHEERS-Studie untersuchte auf Basis der zuvor erfolgten einarmigen Phase-I-Studie die Verstärkung der antitumorösen Immunantwort durch die Kombination von Stereotaxie (SRT) mit Immuncheckpointinhibitoren (ICI) bei Patienten mit fortgeschrittener Tumorerkrankung. Das primäre Ziel war, das progressionsfreie Überleben (PFS) durch verstärkte Antigenfreisetzung im Sinne des abskopalen Effekts zu erhöhen [1].

## Patienten und Methodik

Diese multizentrische, unverblindete, randomisierte Phase-II-Studie wurde an 5 Einrichtungen in Belgien durchgeführt. Eingeschlossen wurden Patienten ≥ 18 Jahre mit fortgeschrittenen Tumoren (HNSCC, malignes Melanom, NSCLC, RCC, UC), Karnofsky-Index ≥ 70 %, Eignung für eine ICI-Therapie und mindestens einer extrakraniellen strahlentherapeutisch behandelbaren Metastase. Ausschlusskriterien waren eine weitere Tumorerkrankung mit Progress oder Therapienotwendigkeit, unkontrollierte ZNS-Erkrankungen, eine vorbestehende Behandlung mit Immunsuppressiva oder ICI. Die Patienten wurden 1:1 unverblindet gemäß Histologie und Tumorlast (≤ 3/≥ 3 Tumorläsionen) in den Kontrollarm mit alleiniger Anti-PD-1/PD-L1-Immuntherapie nach gängigem Standard und den Studienarm mit zusätzlicher SRT ad 3 × 8 Gy, appliziert jeden zweiten Tag für bis zu 3 Tumorherde, randomisiert. Als ICI verwendet wurden Nivolumab, Pembrolizumab und Atezolizumab. Bei Applikation in jeder 2. oder 3. Woche erfolgte die SBRT vor dem 3. Zyklus, während diese bei Applikation alle 4 Wochen vor dem 2. Zyklus erfolgte. Eine palliative Bestrahlung (1 × 8 Gy, 5 × 4 Gy) zur Symptomminderung war in beiden Armen erlaubt. Der primäre Endpunkt war das PFS, sekundäre Endpunkte waren das Gesamtüberleben (OS), die objektive Ansprechrate (ORR) und die lokale Kontrollrate (LCR) gemäß iRECIST-Kriterien. Toxizität wurde nach CTCAE-Kriterien bewertet. Kontrollbildgebungen erfolgten alle 10–12 Wochen oder vorab, sofern klinisch indiziert.

## Ergebnisse

Zwischen März 2018 und Oktober 2020 wurden nach Ausschluss von 3 Patienten bei Widerruf der Zustimmung insgesamt 96 Patienten in die Studie eingeschlossen. Proportional für Demografie und krankheitsspezifische Charakteristika erfolgte die Randomisierung in den Kontrollarm (*n* = 52) und den Studienarm (*n* = 47). Über 70 % der Patienten wiesen mehr als 3 Tumorläsionen auf, 18 % der Patienten im Studienarm erhielten eine Bestrahlung aller aktiven Tumorbefunde. Die häufigsten Lokalisationen waren primäre Lungenherde und Lymphknotenmetastasen. Drei Viertel der Patienten hatten bereits zuvor eine Systemtherapie erhalten, und > 50 % der Patienten im Kontrollarm erhielten vor Studieneinschluss eine Radiatio. Das mediane PFS (2,8 vs. 4,4 Monate) war statistisch nicht signifikant (HR 0,95; 95 %-CI 0,58–1,53; *p* = 0,82), ebenso das OS (11 vs. 14 Monate) nach Abschluss der Beobachtungszeit (HR 0,82; 95 %-CI 0,48–1,41; *p* = 0,47). Es konnte kein Unterschied der Therapieeffizienz zwischen vordefinierten Gruppen der Krankheitslast oder Tumortypen festgestellt werden. Die ORR (22 % vs. 27 %) war ebenfalls nicht signifikant, die LCR betrug 75 % der bestrahlten Läsionen. 8 % und 16 % erreichten eine iCR (iRECIST-Komplettremission), dies war jedoch nur bei Patienten mit malignem Melanom und Urothelkarzinom der Fall. Die Toxizität in den beiden Armen war hierbei vergleichbar (Grad 3 79 % vs. 78 %, Grad 4 18 % vs. 18 %) ohne das Auftreten von Grad-5-Toxizität. Fatigue und Pruritus waren die häufigsten Nebenwirkungen, bis auf gastrointestinale Toxizität, die in beiden Gruppen gleich war.

## Schlussfolgerung der Autoren

Es konnte kein signifikanter Unterschied hinsichtlich PFS und OS zwischen den beiden Armen gezeigt werden. Die Kombination von ICI und SRT zeigte jedoch innerhalb der Studie keine erhöhte Toxizität.

## Kommentar

In dieser bis dahin größten unverblindeten zweiarmigen Studie zur Kombination von SRT und ICI konnte die Effizienz der Kombinationstherapie an einer Reihe von Tumorentitäten untersucht werden. Die SRT erlaubt durch die präzise Applikation hoher Strahlendosen insbesondere bei limitierter Tumorlast eine hohe lokale Kontrollrate bei guter Verträglichkeit. So konnte bei der Bestrahlung aller vorhandenen Tumorläsionen bereits ein verbessertes OS im Vergleich zur alleinigen palliativ-symptomatischen Bestrahlung gezeigt werden [2]. In dieser Studie wurden nur bei acht Patienten alle Tumorläsionen behandelt. Eine Behandlung mit ICI wirkt hingegen systemisch, jedoch hinsichtlich des längerfristigen Therapieansprechens limitiert und in Kombination mit anderen Therapeutika nebenwirkungsträchtig [3, 4].

Zwar konnte weder eine Verbesserung des PFS noch des OS im Studienarm gezeigt werden, jedoch brachte die Kombination der beiden Therapien auch keine erhöhte Toxizität. Die prospektiven Daten decken sich so auch mit bereits vorliegenden Daten einer Metaanalyse [5], die die Kombination von ICI und EGFR-TKI mit kranieller sowie extrakranieller Bestrahlung als gut verträglich wertete. Lediglich die SRT-induzierte schwere Toxizität war dort bei kranieller SRT seltener als bei extrakranieller (6 % vs. 9 %).

Die Anzahl bestrahlter Metastasen, die Heterogenität der bestrahlten Organe mit der Diversität freigesetzter Antigene sowie die prognostisch ungünstige Bestrahlung hämatopoetischer Organe und großer Gefäße mit konsekutiver radiogener Lymphopenie könnten Einfluss auf das therapeutische Ansprechen haben. Die methodisch limitierte Subgruppenanalyse zeigte einen Lymphozytenabfall im Vergleich von Studien- zu Kontrollarm nur bei thorakaler SRT. Insbesondere Lebermetastasen zeigen sich beim NSCLC und malignen Melanom als prognostisch ungünstig mit sowohl lokal als auch systemisch verminderter CD8^+^-T-Zell-Infiltration von Metastasen und so zu erwartend reduziertem Ansprechen auf ICI [6].

Computer- und KI-gestützte Verfahren zur quantitativen Bildgebungsauswertung von Biomarkern wie „CD8 radiomics“ könnten in Zukunft dazu dienen, die Tumorheterogenität und das klinische Ansprechen auf Immuntherapie zu prognostizieren. So zeigen erste Daten, dass Läsionen mit hohem CD8-Scoring mit signifikant besserem Tumoransprechen auf ICI und so verbessertem PFS, OS und „out-of-field response“ im Sinne eines abskopalen Effekts assoziiert waren [7].

Ebenso sollten die Sequenz der SRT zur ICI-Gabe wie auch die Dosisfindung weiter untersucht werden, da zwar die Toxizität sich in den meisten Kombinationsbehandlungen nicht signifikant erhöht [8], jedoch ein Unterschied im Therapieansprechen unklar bleibt. In dieser Studie war die Bestrahlungsdosis durch Zulassung palliativer Konzepte nicht homogen und orientierte sich mit der Dosierung von 3 × 8 Gy an Untersuchungen an humanen und muralen Tumorzelllinien [9]. Die Dosis zielt auf die Induktion kritischer Doppelstrangschäden bei gleichzeitig verminderter Expression von DNA-Exonuklease ab. Diese hemmt den Abbau zytosolischer DNA und interferiert so in einem kritischen „pathway“ zur CD8+-T-Zell-Stimulation.

### Fazit

Die CHEERS-Studie konnte keinen Nachweis einer statistisch signifikanten Verbesserung des PFS und OS durch die Kombination von ICI und STR zeigen; ein zuverlässiger Nachweis des abskopalen Effekts steht so weiterhin aus. Das Ganze bleibt nichtsdestotrotz eines der spannendsten und vielversprechendsten Felder in der modernen onkologischen Komplexbehandlung, da Untersuchungen zu prädiktiven Markern laufen und immer neue Daten alternative Therapiekonzepte ins Spiel bringen, beispielsweise die Kombination von STR und Nivolumab im Frühstadium des NSCLC [10].

*Tobias Mohr, Homburg/Saar*
